# Single-cut-Derotationsosteotomie am distalen Femur zur Korrektur von Torsion und frontaler Achse

**DOI:** 10.1007/s00064-024-00844-y

**Published:** 2024-03-27

**Authors:** Florian B. Imhoff, Mathieu Trierweiler

**Affiliations:** 1 Orthopädie und Traumatologie, Standort Bethesda Spital, Universitätsspital Basel, Basel, Schweiz Gellertstrasse 144, 4052; 2 Kantonsspital Baselland, Liestal, Schweiz

**Keywords:** Distale femorale Derotationsosteotomie, Single-cut-Osteotomie, 3D-Korrektur, Patientenspezifische Schnittblöcke, Distal femoral derotational osteotomy, Single cut osteotomy, 3D correction, Patient-specific cutting guides

## Abstract

**Operationsziel:**

Die Rotationsosteotomie bedingt eine komplette Durchtrennung des Knochens zur Korrektur der Maltorsion. Eine zusätzliche Korrektur der frontalen Achse kann durch eine definierte schräge Sägeebene der Osteotomie erzielt werden. Der direkte flächige Knochenkontakt wird mit einer winkelstabilen Osteosyntheseplatte fixiert.

**Indikationen:**

Symptome wie anteriorer Knieschmerz, Inwardly-pointing-knee-Syndrom, laterale Patella(sub)luxation, laterale Patellahyperpression sind typische Beschwerden, welche in Kombination mit klinisch erhöhter femoraler Innenrotation und radiologisch erhöhter femoraler Antetorsion zur Indikation der Derotationsosteotomie führen.

**Kontraindikationen:**

Kontraindikationen für die o. g. Derotation bestehen bei vermehrter Hüftaußenrotation versus Innenrotation, erhöhter femoraler Torsion aber keine vermehrte Hüftinnenrotation, Malcompliance wie Unfähigkeit der Stock-Teilbelastung. Zudem stellen generelle Risiken, die zu einer verzögerten Knochenheilung führen wie Nikotinabusus und Adipositas, relative Kontraindikationen dar; ebenso wie eine bereits bestehende patellofemorale Arthrose oder die Einnahme von Glukokortikoiden und Immunsuppressiva.

**Operationstechnik:**

Es kann ein lateraler oder wahlweise medialer Zugang zum distalen Femur zur Darstellung des Knochens mit Eva-Haken gewählt werden. Die Verwendung von patientenspezifischen Schnittblöcke geben das geplante Ausmaß der Derotation und Ebene der Schnittführung sehr präzise vor. Durch eine definierte Single-cut-Schnittebene kann zusätzlich die frontale Achse korrigiert/verbessert werden. Eine zusätzliche biplanare Schnittführung mit anteriorem Wedge erhöht die intraoperative Stabilität und generiert eine größere Fläche für die Konsolidierung.

**Weiterbehandlung:**

Aufgrund des extramedullären Kraftträgers ist eine Teilbelastung mit 15–20 kg an Stöcken für 6 Wochen empfohlen mit jedoch freier Beweglichkeit des Kniegelenks

**Ergebnisse:**

Die Literatur beschreibt signifikant erhöhte Patientenzufriedenheit in Bezug auf Patellastabilität und Kniefunktion nach Kombinationseingriffen mit Derotationsosteotomie. Mit der Verwendung von PSI-Schnittblöcken ist die Genauigkeit der Osteotomie und der 3‑dimensionsalen Korrektur sehr hoch. Die delayed-union-Rate liegt bei ca. 10 %.

## Abstract (en)

**Objective:**

A rotational osteotomy requires a complete cut of the bone in order to correct maltorsion. An additional correction of the frontal axis can be achieved via an oblique cut of the bone. The osteotomy with bone to bone contact is fixed with an angle stable plate.

**Indications:**

Symptoms such as anterior knee pain, inwardly pointing knee syndrome, lateral patellar subluxation or dislocation, lateral patellar hypercompression syndrome are a common indication for derivational osteotomy if clinically increased femoral internal rotation and radiologically increased femoral antetorsion is detected.

**Contraindications:**

Increased hip external rotation versus internal rotation, increased femoral torsion but no increased internal hip rotation, malcompliance, inability for partial weight bearing, risk of delayed union (nicotine abuse and obesity) as well as patellofemoral arthritis and systematic glucocorticoids, immunosuppressants are (relative) contra-indications.

**Surgical technique:**

A lateral or optionally medial approach to the distal femur and exposure of the bone with Eva hooks for the osteotomy is done. The use of patient-specific cutting blocks accurately specify the planned extent of derotation and level of incision. A defined oblique cutting plane of the single-cut osteotomy and derotation will additionally correct/change frontal axis. An additional biplanar osteotomy with an anterior wedge increases intraoperative stability and generates a larger bone contact area for consolidation.

**Postoperative management:**

With the use of an extra medullary fixation device partial weight bearing with 15–20 kg with crutches up to 6 weeks is required, but no restriction on knee movement is given.

**Results:**

The literature shows significantly improved patient satisfaction regarding patellofemoral stability and knee function. With the use of patient-specific cutting guides, high accuracy of the osteotomy and 3‑dimensional correction can be achieved, while delayed union rate is up to 10%.

## Vorbemerkungen

Eine erhöhte femorale Antetorsion ist ein wichtiger Risikofaktor für die patellofemorale Instabilität und das vordere Knieschmerzsyndrom im Adoleszentenalter und bei Erwachsenen [2, 7]. Durch eine Derotationsosteotomie wird das distale Femur nach außen gedreht und ist eine zuverlässige Option, um diese Torsionspathologie im Rahmen der patellofemoralen Instabilität in Kombination mit zusätzlichen Bandstabilisierungen zu korrigieren [6]. In der Literatur gibt es verschiedene Beschreibungen der Technik: entweder über einen medialen oder lateralen Zugang, Lage und Einstellung der Osteotomie [1, 3, 12]. Allerdings kann durch eine Rotation und einen leicht schrägen Schnitt zur mechanischen Achse eine ungewollte Veränderung der frontalen und sagittalen Achseinrichtung entstehen. Im schlimmsten Fall kann durch eine Korrektur der femoralen Maltorsion bei patellofemoraler Instabilität gleichzeitig ein vermehrter Valgus entstehen, was bei dieser Grundproblematik der lateral luxierenden Patella die Symptome sogar verstärken kann [13].

In einem Berechnungsmodell über die 3‑dimensionalen Korrekturebenen konnte gezeigt werden, wie schräg eine Sägeebene bei einer Single-cut-Osteotomie sein muss, um bei gegebenem Derotationswinkel eine entsprechende Veränderung der frontalen Achse zu erreichen [5].

Hier ist wiederum die Implementierung der Planung am 2D-Röntgenbild in die Praxis und an den intraoperativ dargestellten Knochen sehr schwierig und lässt eine gewisse Varianz bzw. Ungenauigkeit zu. Hierfür eignen sich patientenspezifische Schnittblöcke extrem gut, welche zum einen die geplante Korrektur der Drehung/Rotation und zum anderen auch die schräge Sägeebene vorgeben [9]. Im Folgenden wird die Single-cut-Osteotomie am distalen Femur mit und ohne Schnittblöcke gezeigt.

## Operationsprinzip und -ziel

**Figure 1:**
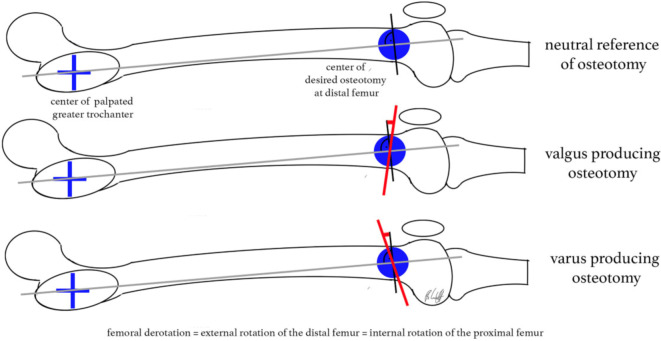

Nach kompletter Osteotomie am distalen Femur wird der distale Anteil um die Längsachse des anatomischen Schafts rotiert. Der Zugang kann von lateral oder medial erfolgen. Bei grundlegend erhöhter femoraler Antetorsion wird das distale Fragment nach außen gedreht, um eine Derotation zu erzielen. Gleichzeitig kann durch eine schräge Osteotomie zum Schaft im Rahmen der Drehung auch eine Veränderung/Korrektur der frontalen Achse erzielt werden (Abb. 1). Nach Single-cut-Osteotomie bleibt ein direkter Knochenkontakt erhalten, was mit einer winkelstabilen Platte (von lateral oder medial) fixiert wird. Um eine zusätzliche Auflagefläche der Osteotomie zu erreichen, kann ein anteriores Schild in Keiltechnik generiert werden.

## Vorteile

- Direkter Knochenkontakt ohne ossären Gap
- Biplanare Schnittführung mit anteriorem Schild führt zu erhöhtem Knochenkontakt und Stabilität der Osteotomie.
- Gute anatomische Übersicht über einen lateralen Subvastus-Zugang
- Geringe Zugangsmorbidität
- Gefäße und Nerven können sicher weggehalten und geschützt werden.
- Standard-Plattensysteme können verwendet werden.
- PSI-Schnittblöcke verbessern die Genauigkeit.

## Nachteile

- Keine sofortige Vollbelastung möglich im Vergleich zu Marknagel-Fixateuren
- Traktus-Irritation durch Plattenlage sehr häufig, Folgeoperation mit Plattenentfernung notwendig
- Zusatzeingriffe wie MPFL-Rekonstruktion erschwert bei lateraler und auch medialer Plattenlage (Röntgenbild für optimalen Eintrittspunkt und Durchzug-Shuttlefäden)

## Indikationen

Femorale Maltorsion:

- Chronische patellofemorale Instabilität insbesondere im Revisionsfall, „inwardly-pointing knee“, anteriore Knieschmerzen, laterale Patella-Hyperpression,
- klinisch erhöhte Hüft-Innenrotation > 50° und verringerte Hüft-Außenrotation (< 20°),
- auffälliger Innenrotations-Adduktions-Gang, erhöhter Dynamic-Q-Angle,
- femorale Torsionswerte (MRT/CT) erhöht je nach Messmethode: Schneider et al. (MRT) > 25°, Waidelich et al. (CT) > 40°,
- posttraumatische und/oder iatrogen entstandene femorale Maltorsion, entsprechende Messmethode mit axialer Schnittbildgebung.

## Kontraindikationen

- Klinisch keine erhöhte Hüft-Innenrotation trotz erhöhter femoraler Torsionswerte (es würde ein Außenrotationsgang resultieren ⇒ Acetabulumversion beachten)
- Rauchen und auch Adipositas sind relative Kontraindikationen, da mit verzögerter Knochenheilung zu rechnen ist
- Malcompliance in der Nachbehandlung (Teilbelastung nicht möglich)
- Offene Wachstumsfugen ist eine relative Kontraindikation.
- Schwere Patellofemoralarthrose als relative Kontraindikation

## Patientenaufklärung

- Das erreichbare Korrekturziel der Derotation ist laut Literatur mit ± 2° angegeben
- Verzögerte Knochenheilung und Pseudarthrose
- Revisionseingriff zur sekundären Stabilisation des medialen Kortex bei Korrekturverlust oder Schraubenlockerung
- Intraoperative Gefäß- und Nervenverletzung mit weiterer sofortiger Intervention
- Adaptation des Gangbilds dauert mindestens 6 Monate und verbessert sich bis zu 12 Monate postoperativ
- Bei neurologischer Grunderkrankung ist der muskuläre Aufbau postoperativ beeinträchtigt und verlängert.

## Operationsvorbereitungen

- Planung anhand von Röntgen-Ganzbeinaufnahme (ist zusätzlich eine frontale Korrektur notwendig?)
- Axiale Bildgebung (MRT oder CT) zur Bemessung der Torsion und Entscheid zum Korrekturziel
- 3D-Ganganalyse sinnvoll
- Lagerung im elektrischen Beinhalter mit Blutsperren-Manschette am Oberschenkel soweit proximal wie möglich

## Instrumentarium

- Eva-Haken, K‑Drähte (Stärke 2,0 mm oder 1,6 mm) für die Vorgabe der Osteotomieebene, Sägeblatt (z. B. Gomina-Sägeblatt: Stärke 0,9 mm, Länge 90 mm)
- Winkelmesser, Fixateur externe Grundmaterial mit Verriegelungsbolzen und Schanz-Pins
- Patientenspezifische Schnittblöcke (PSI)
- Winkelstabile Platten, Typ Fixateur interne

## Anästhesie und Lagerung

- Standardlagerung in Rückenlage. Blutsperrenmanschette wird soweit wie möglich proximal angelegt, jedoch nicht aktiviert (intraoperative Koagulation), elektrischer Beinhalter hat sich bewährt für eine feste proximale Fixierung, sodass bei Repositionsmaneuvern nach kompletter Osteotomie nur das distale Fragment manipuliert und gehalten werden muss.
- Spinalanästhesie sowie Allgemeinanästhesie möglich, ggf. zusätzlicher Schmerzkatheter oder „single-shot“ N. femoralis
- Perioperative Antibiotikaprophylaxe Cefuroxim intravenös gewichtsadaptiert
- Cyclokapron 1 g intravenös perioperativ

## Operationstechnik

**Figure 2:**
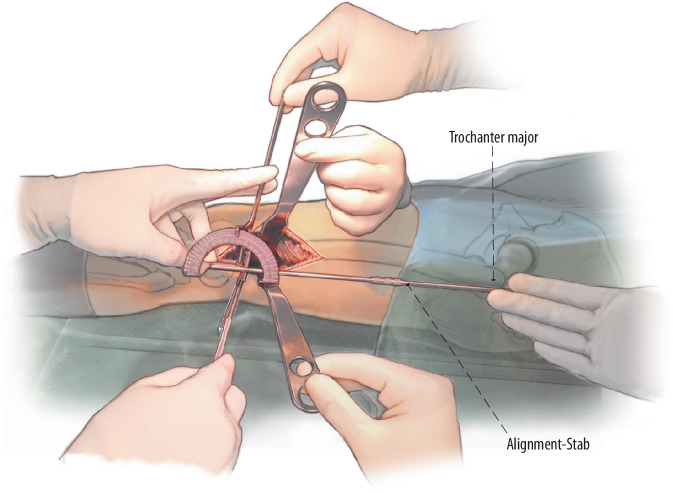

**Figure 3:**
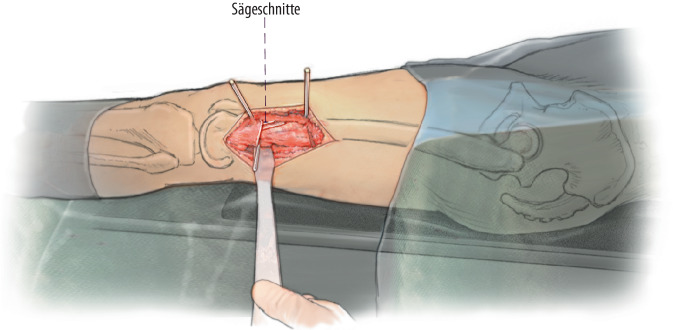

(Abb. 2, 3, 4 und 5)

**Figure 4:**
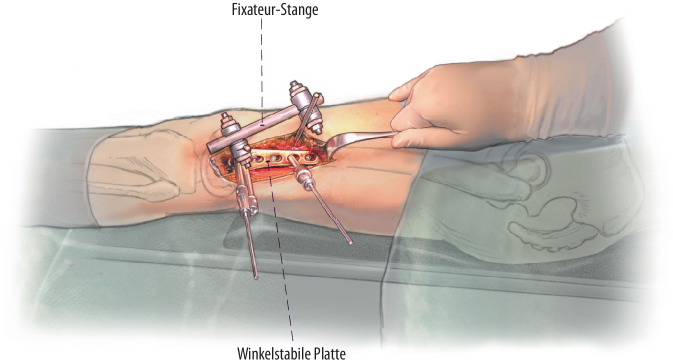

**Figure 5:**
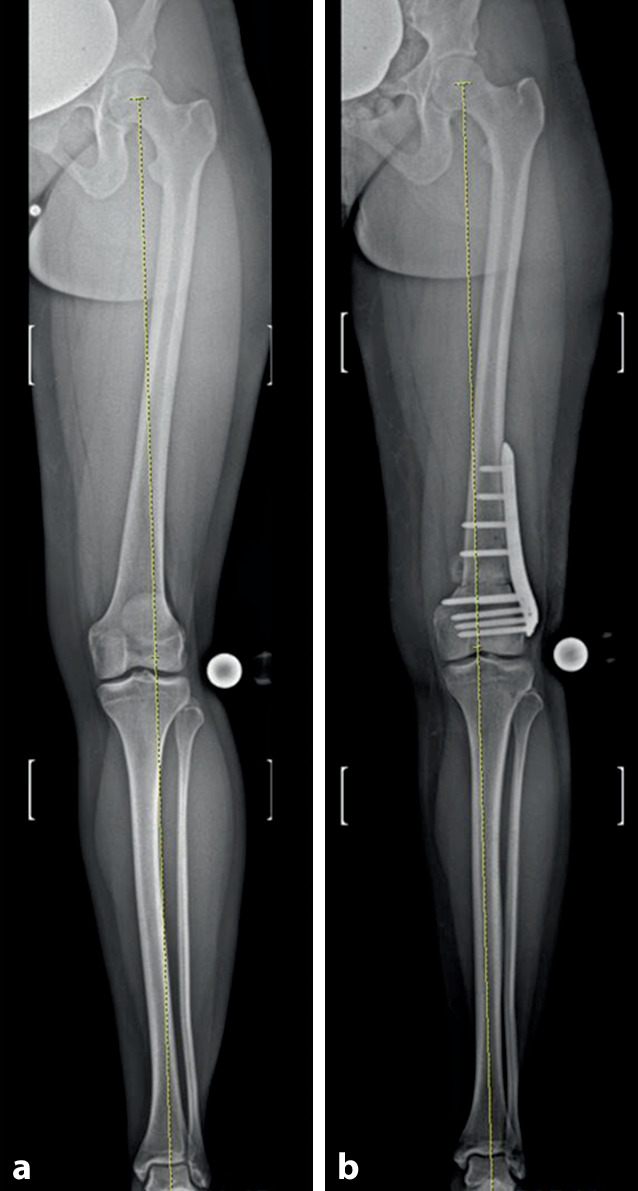

## Besonderheiten

**Figure 6:**
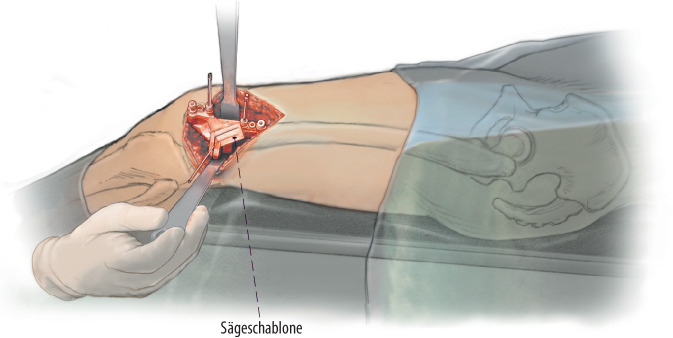

**Figure 7:**
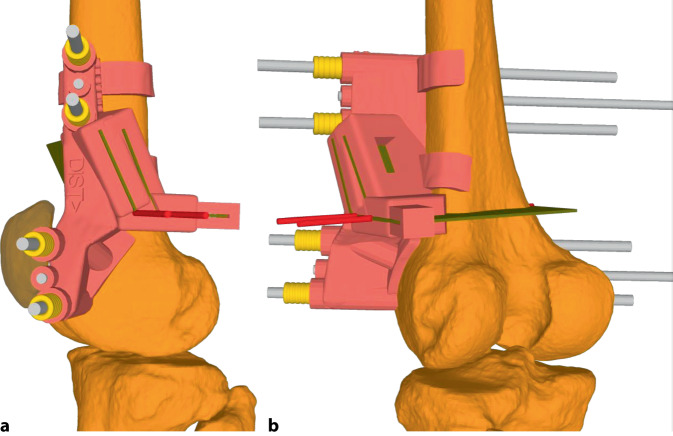

**Figure 8:**
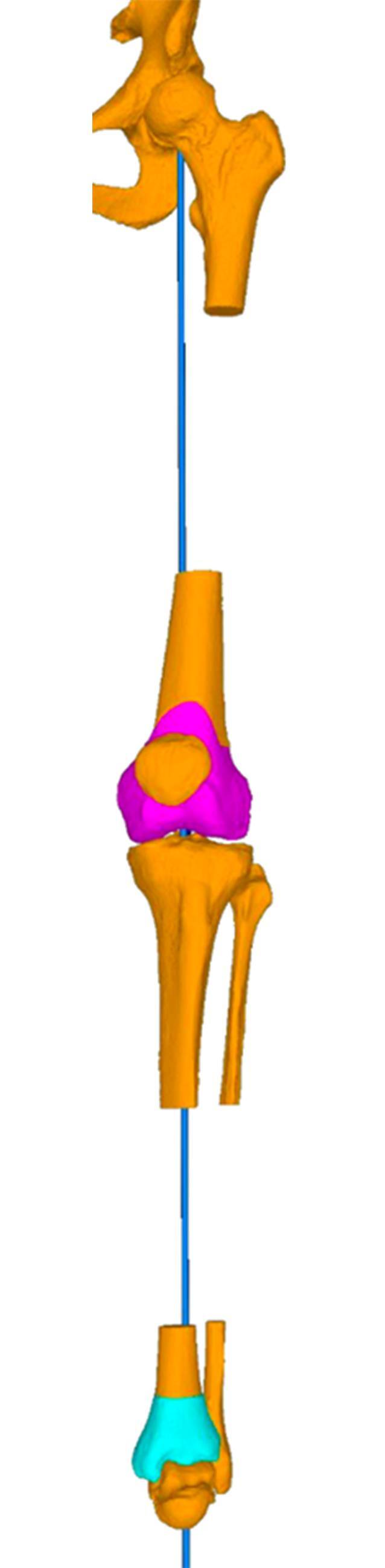

**Figure 9:**
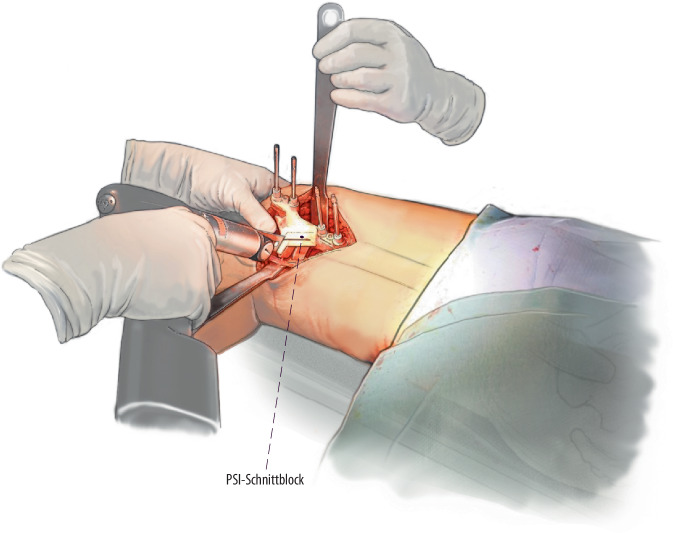

(Abb. 6, 7, 8, 9, 10 und 11)

**Figure 10:**
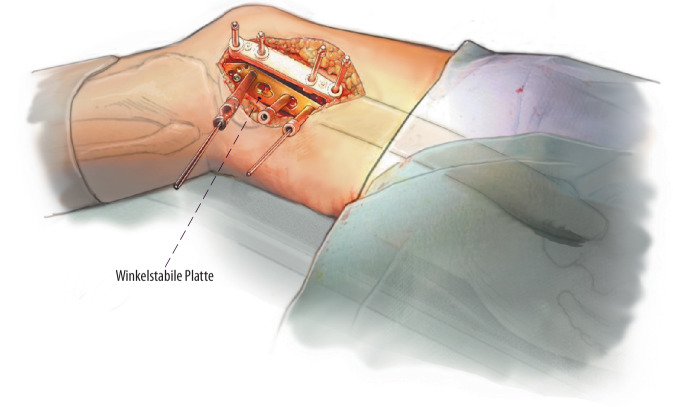

**Figure 11:**
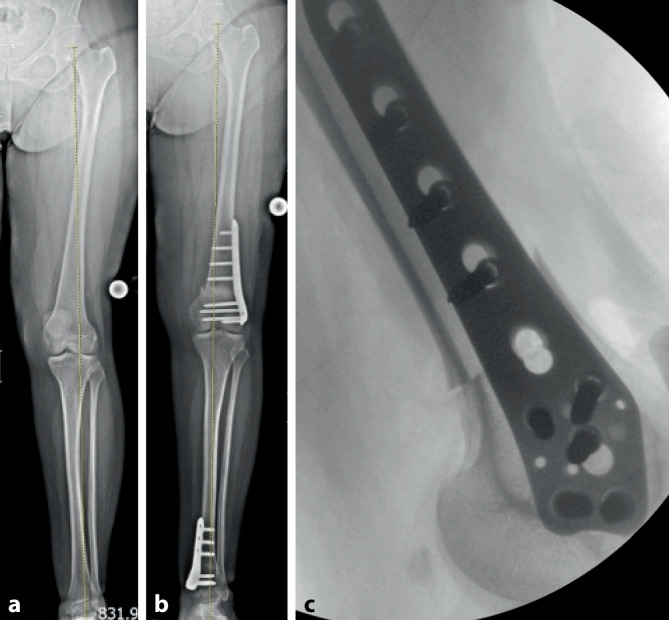

## Postoperative Behandlung

- Teilbelastung 15–20 kg wegen Plattenosteosynthese für 6 Wochen, Thromboseprophylaxe in dieser Zeit (mit z. B. niedermolekularem Heparin s.c.)
- Freie Beweglichkeit des Kniegelenks
- Mobilisation unter physiotherapeutischer Anleitung mit Gehstöcken als Ziel, ggf. zusätzliche Gehhilfe (Rollator, Rollstuhl)
- Passive Bewegungsschiene im Liegen ab 1. postoperativer Tag. Elektrostimulation der Oberschenkelmuskulatur zur Verminderung der zu erwartenden Muskelatrophie
- Adäquate Schmerztherapie, optional Schmerzkatheter möglich bis 2 Tage postoperativ
- Elastokompressive Wickelung ohne Gipsschiene oder Gipsverband, Entfernung Drainagematerial 2. postoperativer Tag, Wechsel auf Kompressionsstrumpf im Verlauf
- Klammer/Fadenentfernung 12–14 Tage postoperativ
- Erste radiologische Nachkontrolle 6 Wochen postoperativ: Röntgenaufnahme Knie a.-p./lateral
- Nach 6 Wochen Übergang zur möglichen Vollbelastung schmerzadaptiert, Benutzung der Gehhilfen (Stöcke, Rollator) bis erlangte und sichere Vollbelastung. Zunehmende Kräftigungsübungen und Beinachsentraining physiotherapeutisch instruiert.
- Nächste radiologische Verlaufskontrolle 3 Monate postoperativ mit Zielsetzung erlangte Vollbelastung ohne Gehhilfe. Röntgenaufnahme Knie a.-p./lateral und Ganzbeinaufnahme Orthoradiogramm
- Weitere Nachkontrollen klinisch und bei Bedarf radiologisch 4,5 Monate, 6 Monate, 9 Monate, 1 Jahr postoperativ mit zusätzlicher Ganganalyse und biomechanischer Testung zum präoperativen Vergleich. Anpassungszeit des Gangbilds kann bis 1 Jahr postoperativ brauchen.
- Bei Traktus-Irritation durch einliegendes Plattenlager, Entfernung möglich bei vollständiger radiologischer Konsolidierung

## Fehler, Gefahren, Komplikationen und ihre Behandlung

- Normalerweise ist ein interner Plattenfixateur (z. B. Synthes Tomofix) medial oder lateral ausreichend, bei Korrekturverlust und Insuffizienz der Osteosynthese (weniger wie 3 bikortikale Schrauben proximal) ist eine Doppelplatten-Osteosynthese zu erwägen.
- Marknagel-Fixierung ist eine gute Alternative und ist sofort belastungsstabil. Der Nachteil liegt in der zusätzlichen Zugangsmorbidität und im Rahmen von gelenknahen Osteotomien schlechteren Verankerungsmöglichkeiten der Bolzen, was wiederum zu einem Korrekturverlust führen kann.
- Komplikationen der Plattenosteosynthese sind die verzögerte Knochenheilung und Pseudarthrosenbildung. Normalerweise ist eine volle Durchbauung radiologisch nach 4–6 Monaten erzielt. Eine CT-Bildgebung kann abwägen, ob ein Stabilitätsproblem (hypertrophe Pseudarthrose) vorliegt und eine zusätzliche Stabilität über eine zweite Platte notwendig ist.
- Fehlerhafte Inklination der Sägeebene und/oder zu geringe/zu viel Derotation können das Korrekturziel in der frontalen Achse wesentlich beeinflussen und müssen daher möglichst exakt (innerhalb von 1–2° Toleranz) durchgeführt werden.
- Die Verletzung von arteriellen Gefäßen, insbesondere A. femoralis/poplitea ist eine schwerwiegende Komplikation, die einer unmittelbaren Intervention durch einen Gefäßchirurgen bedarf. Im eigenen Vorgehen wird die Osteotomie ohne Blutsperre durchgeführt, um im Notfall entsprechend reagieren zu können und Zeit zu gewinnen.
- Verletzungen von Nerven (N. ischiadicus)

## Ergebnisse

In einer kürzlich erschienen Studie, bei der der Erstautor mitwirkte, wurde das funktionelle und radiologische Ergebnis nach distaler femoraler Derotationsosteotomie bei Patienten mit patellofemoraler Instabilität und einer erhöhten femoralen Antetorsion untersucht [4]. Eingeschlossen wurden Patienten mit Operationszeitpunkt zwischen 2011 und 2018 und einem Follow-up von mindestens 24 Monaten. Prä- und postoperativ wurden die visuelle Analogskala (VAS) für Schmerzen, Kujala-Score, Lysholm-Score, subjektive Knieform des International Knee Documentation Committee (IKDC) und Tegner Activity Scale (TAS) Scores ausgewertet. Zudem wurden Röntgen-Ganzbeinaufnahmen vor und nach der Operation durchgeführt sowie jeweils eine MRT-Bildgebung (Hüfte-Knie) zur Torsionsmessung des Femurs. Insgesamt wurden 27 Patienten (30 Knie) eingeschlossen. In 25 Fällen (83,3 %) wurden begleitende Eingriffe zur patellofemoralen Instabilität durchgeführt. Die femorale Antetorsion war signifikant reduziert (28,2 ± 6,4° vs. 13,6 ± 5,2°; *p* < 0,05). In der Gruppe mit zusätzlicher Achskorrektur (14 Fälle [46,7 %]) wurde eine Achskorrektur von präoperativ 2,4 ± 1,2° Valgus vs. 0,3 ± 2,4° Valgus (*p* < 0,05) gemessen.

Die klinischen Outcome-Parameter zeigten zum einen eine signifikante Schmerzreduktion (VAS für Schmerzen: 2,0 [1,0–5,0] vs. 0 [0–1,0]; *p* < 0,05) und eine signifikante Verbesserung der Kniefunktion (Kujala-Score: 55,6 ± SD 13,6 vs. 80,3 ± 16,7; *p* < 0,05; Lysholm-Score: 58,6 ± 17,4 vs. 79,5 ± 16,6; *p* < 0,05; IKDC: 54,6 ± 18,7 vs. 74,1 ± 15,0; *p* < 0,05) und eine Zunahme der sportlichen Aktivität (TAS: 3,0 [3,0–4,0] vs. 4,0 [3,0–5,0]; *p* = n. s.) im mittleren Beobachtungszeitraum von 38 Monaten. In einem Fall kam es 70 Monate nach der Operation zu einer Patellareluxation.

Eine Studie zur Genauigkeit der Derotationen mit patientenspezifischen Schnittblöcken zeigte eine Abweichung von 4,8° ± 3,1°.

In der Nachuntersuchung von 40 Knien nach Derotation am distalen Femur mit PSI wurde die Genauigkeit mit 1,5 ± 1,4° für femorale Anteversion (axiale Torsionskorrektur) und 0,9 ± 0,9° für die frontale Achse, gemessen am lateralen distalen Femurwinkel, angegeben [11]. In anderen Studien wurde die Komplikationsrate bzgl. Wundheilungsstörung mit 4 % angegeben und die „non-union rate“ mit bis zu 10 % [10]. Im eigenen Vorgehen wird die Indikation zur zusätzlichen medialen Platte gestellt, falls nach 3–4 Monaten keine schmerzfreie Belastung möglich ist oder eine hypertrophe Kallusbildung vorliegt.
